# Supplementary material for: Distribution of ESBL/AmpC-Escherichia coli on a Dairy Farm
Source: Antibiotics (Basel). 2022 Jul 13;11(7):940. doi: 10.3390/antibiotics11070940 (PMC9311582; doi:10.3390/antibiotics11070940)
Supplement: Supplementary file 1 [file antibiotics-11-00940-s001.zip › antibiotics-1816236-Supplementary.pdf]

**Supplement File S1.** Questionnaire of the study Prevalence and risk factors for the occurrence of ESBL *E. coli* in pre-weaned dairy calves and their dams on large dairy farms in Germany - a cross-sectional study

<u>Data of the participants</u>

---

---

**<u>General Data</u>**

**x** conventional farming

○ organic farming (since ________________)

■Are the other livestock husbandries within a radius of 3 km?

**x** yes, <u>dairy cattle (species)</u>

○ no

■Frequently buy-in of cattle?

○ yes

**x** no

If yes, how often?

○ more than once a month

○ every 1-3 months

○ less than every 3 months

Information of the origin farm/s

○ only 1 origin farm

○ > 1 fixed origin farms

○ > 1 changing farms

■Outsourced heifer rearing?

**x** yes

○ no

If yes, in

**x** another location of the own farm

○ an external farm

○ heifers are raised at more than 1 location ______ (number)

○ there are external animals at this location

■Which animals feeds are used for calves and cows:

**x** in-house basic forage: grass silage, corn silage, hay, straw

○ in-house substrates_______________________________________________

○ external basic forage__________________________________________

**x** external substrates: rapeseed extraction meal, soybean meal, beet pulp, feed fat, minerals, calf pellets

○ bought in forage comes from a single farm

**x** bought in forage comes from different farms

How often do the producers change?

○ > 1 x / month

○ every 1-3 months

**x** less than every 3 months

■Fertilization of the fields that are used for the feed/bedding material production

**x** in-house slurry

**x** external slurry

■Does an in-house biogas reactor exist?

**x** yes

○ no

If yes, do you use in-house material exclusively?

**x** yes ○ no, ____________________________________________________________

Are the fields that are used for feed production or bedding material fertilized with the outcome of the biogas plant, that were fed with external slurry/manure?

○ yes ○ no

How many external husbandries are involved?

**x** never

○ 1-3

○ more than 3

How often do external husbandries change?

**x** never

○ more than every 3 months

○ less than every 3 months

Which species are kept on external husbandries?

○ poultry

○ pigs

○ ___________________

■Which people have contact with the livestock animals?

**x** personnel

**x** veterinarian

**x** family members

**x** visitors (e. g. providers, craftsman)

Do this people have contact to other animals?

**x** yes, to dogs, cats, horses, poultry, cattle (species)

○ no

■Do you perform pest control?

**x** yes, against rats

○ no

**Calving management**

Housing: **x** single pen **x** group pen

**x** straw bedding ○ ___________

**Hygiene-management:**

Cleaning frequency?

○ after each birth

○ more than once a week

**x** less than once a week

How long do calves stay with their dams after calving?

**x** <1h

**x** 1-3h

○ >3h

Co-husbandry of calving and dry cows?

○ yes, in more than half of the calvings

**x** yes, in less than half of the calvings

○ no

Co-husbandry of calving and sick cows?

○ yes, in more than half of the calvings

○ yes, in less than half of the calvings

**x** no

**<u>Calves up to 28d</u>**

**Housing:**

**x** single Igloo (100%) ○ single pen (____%)

○ group Igloo (____%) ○ group pen (____%)

■Change of the housing:

○ 2 weeks

○ no change

**x** <u>about after 4 weeks</u>

New housing:

○ group igloo (____%)

**x** group pen (100%)

■Floor:

**x** concrete floor

○ natural floor

■Bedding material and frequency:

**x** straw

**x** daily bedding

○ bedding if necessary

■Direct contact to other calves:

**x** yes

○ no

■Number of calves per group:

**x** calve-feedlot Ratio >1:1

○ calve-feedlot Ratio <1:1

■Direct contact to other animals

○ yes

**x** no

○ ____________________________________________ (species)

■All-in-all-out-principle

○ yes

**x** no

If yes,

○ all calves

○ not for all calves (____%)

**Feeding:**

■<u>Colostrum feeding:</u>

○ immediately after birth

**x** until 3 h after birth

○ later than 3 h

**x** colostrum of the own dam (**x** milked off ○ directly from the dam)

○ colostrum stored frozen and defrosted

**x** colostrum stored in the refrigerator

○ colostrum is pasteurized

○ colostrum is acidified (with_______________)

**x** the dams have been present on the farm for at least 3 weeks before birth

○ foreign colostrum is occasionally used

origin farm: ___________________________________________________

How often? ________________________ How many calves? _________________)

○ check of colostrum quality

How? ____________________________________

How much colostrum do calves receive? <u>3-4 liters</u>

How?

**x** drench **x** nursing bucket

<u>Further feeding management:</u>

■○ all calves **x** heifer calves

Age: from <u>first day</u> to <u>4 weeks</u>

○ raw milk

○ waste milk (antibiotic residues)

○ waste milk (without antibiotic residues)

**x** milk replacer

**x** 2 x per day, 4 liters (volume)

○ ad libitum

○ milk is pasteurized

○ milk is acidified

Age: From 5 weeks to 8 or 9 weeks

○ raw milk

○ waste milk (antibiotic residues)

○ waste milk (without antibiotic residues)

**x** milk replacer

**x** 2 x per day, At the beginning 4 liters, then the amount is reduced by one liter per week, so that the calves are weaned at about 8 to 9 weeks of age (volume)

○ ad libitum

○ milk is pasteurized

○ milk is acidified

■○ male calves

Age: From __________________ to ___________________

○ raw milk

○ waste milk (antibiotic residues)

○ waste milk (without antibiotic residues)

○ milk replacer

○ __ x per day, _______________(volume)

○ ad libitum

○ milk is pasteurized

○ milk is acidified

Age: From __________________ to ___________________

○ raw milk

○ waste milk (antibiotic residues)

○ waste milk (without antibiotic residues)

○ milk replacer

○ __ x per day, _______________(volume)

○ ad libitum

○ milk is pasteurized

○ milk is acidified

■Feeding practice?

**x** always at fixed times

○ irregularly

○ use of automatic feeder (○ freshly prepared, ○stored)

■How is the calve feeder cleaned and disinfected?

○ no cleaning and disinfection

**x** with water only

**x** with cleaning agent <u>(when reallocating buckets to new calves.)</u>

○ with cleaning and disinfectant

How often is the calf feeder cleaned and disinfected?

○ after each meal

**x** 1 x / day

○ < 1 x / day

■Do calves receive supplementary feed?

**x** yes, roughage from day <u>5. week of life</u>

**x** yes, concentrate from day <u>1</u>

○ no

What is used to prepare the milk replacer?

○ tap water

**x** well water

■Do the calves have free water access?

**x** yes ○ no

○ tap water

**x** well water

■Which people take care of the calves?

**x** number 2-3

**x** trained ○ untrained

○ changing **x** constant

Use of protective clothing?

○ yes

**x** no (Only farm-owned work clothing)

■Use of preventive treatment for calves?

○ yes, ___

**x** no

How do you assess calf health? What are the biggest problems?

Basically, the health is good, the biggest problem is calf diarrhea.

What do you do about it?

Optimization of colostrum supply, clean igloos and plenty of straw as bedding.

Use of treatment schedules for claves?

x yes

○ no

In case of diarrhea, the calves are given an electrolyte drinker. If there is no improvement, Metapyrin is used and long-lasting diarrhea is treated with Synulox or Enrotron.

**Cows**

**Housing systems:**

**x** cubicle housing system (____%) (all lactating cows and dry cows)

**x** deep-bedded straw yard housing system (____%) (dry cows and heifers 2-3 weeks before calving and 1 week after calving.)

o flat floors (____%) o slatted floors (____%)

o high cubicle beds (____%) o low cubicle beds (____%)

**x** straw (____%)

**x** sawdust (____%)

o ___________________________________

o lime (____%)

o pasture (____%)

**Dry-off-management:**

Cows are dried with antibiotic treatment Orbenin(name)

Use of teat sealant?

**x** yes

o no

Cows are dried 55-60 days/weeks bevore birth date.

Use of maternal vaccinations?

o yes, Rota/Corona/E.coli

o yes, ___________________________

**x** no

**Milking routine:**

Udder preparation:

o dry cleaning

o wet cleaning __________________

o pre-dip

o milked by hand

**x** stimulated per brush __________

Use of post-milking treatment

**x** yes, with ______________________

○ dip

**x** spray (automatic)

Use of milking cluster disinfection

**x** yes, with 2% Peracetic acid, automatic

○ no

Use of separate milking parlor for sick cows?

○ yes

**x** no

How is the milking order?

**x** cows with waste milk at the end

**x** cows with high somatic cell count ~~at the end~~ second last

○ mixed order

**Health management:**

Use of preventive treatment for cows?

○ yes, _______________________________________________________ (____%)

**x** no

Use of treatment schedules for cows?

**x** yes

○ no

Is every clinical mastitis treated with antibiotics?

**x** yes, with Taneven, Ubrolexin (____%)

○ no

Other treatment schedules:

If a treatment doesn´t improve the health of the animal, when do you change the antibiotic treatment?

o never (____%)

o after 1 day (____%)

o after 2 days (____%)

**x** after 3 days (____%)

o only after completion of the treatment (____%)

Use of alternative treatment methods, e. g. homeopathy?

o yes, ______________________________________________________ (____%)

**x** no

Housing of the cows producing waste milk?

o in the regular group (____%)

**x** separated with other waste milk cows (____%)

o separated with other cows, that were treated with antibiotics (____%)

**Cleaning and disinfection management:**

How does the cleaning and disinfection proceed?

| Calving pen | Calf housing | Milking parlor |
|---|---|---|
| Cleaner<br><br>**x** we do not use any<br>cleaning agent<br><br>Exposure time<br>o shorter than the manufacturer's instructions<br>o according to the manufacturer's instructions | Cleaner<br><br>o we do not use any<br>cleaning agent<br><br>Exposure time<br>o shorter than the manufacturer's instructions<br>**x** according to the manufacturer's instructions | Cleaner<br><br>o we do not use any<br>cleaning agent<br><br>Exposure time<br>o shorter than the manufacturer's instructions<br>**x** according to the manufacturer's instructions |

| | | |
|---|---|---|
| Is there complete dry-through?<br><br>○ yes<br>○ no | Is there complete dry-through?<br><br>**x** yes<br>○ no | Is there complete dry-through?<br><br>○ yes<br>**x** no |
| Disinfectant<br><br>**x** we do not use a disinfectant<br><br>Exposure time<br>○ shorter than the manufacturer's instructions<br>○ according to the manufacturer's instructions | Disinfectant<br><br>○ we do not use a disinfectant<br><br>Exposure time<br>○ shorter than the manufacturer's instructions<br>**x** according to the manufacturer's instructions | Disinfectant<br><br>○ we do not use a disinfectant<br><br>Exposure time<br>○ shorter than the manufacturer's instructions<br>**x** according to the manufacturer's instructions |
| Use of cleaning and disinfection plan<br><br>○ yes<br>**x** no | Use of cleaning and disinfection plan<br><br>**x** yes<br>○ no | Use of cleaning and disinfection plan<br><br>**x** yes<br>○ no |
| Cleaning agents?<br><br>○ Surfactants<br>○ Acids<br>○ Bases<br>○ Bleaching agents<br>○ Enzymes | Cleaning agents?<br><br>○ Surfactants<br>**x** Acids<br>**x** Bases<br>○ Bleaching agents<br>○ Enzymes | Cleaning agents?<br><br>○ Surfactants<br>**x** Acids<br>**x** Bases<br>○ Bleaching agents<br>○ Enzymes |
| Disinfectants?<br><br>○ Peracetic acid<br>○ Hydrogen peroxide<br>○ Chlorine<br>○ Iodine<br>○ Aldehydes | Disinfectants?<br><br>**x** Peracetic acid<br>○ Hydrogen peroxide<br>○ Chlorine<br>○ Iodine<br>○ Aldehydes | Disinfectants?<br><br>**x** Peracetic acid<br>○ Hydrogen peroxide<br>**x** Chlorine<br>○ Iodine<br>○ Aldehydes |

| | | |
|---|---|---|
| ○ Alcohols<br>○ Phenols<br>○ Nitrogen compounds | ○ Alcohols<br>○ Phenols<br>○ Nitrogen compounds | ○ Alcohols<br>○ Phenols<br>○ Nitrogen compounds |

**Assessment of the herd manager**

What is the biggest health problem of the herd?

---

---

How many animals do you treat with antibiotics per day on average?

**x** <2 per 100 animals treated

○ 2-5 per 100 animals treated

○ 5-10 per 100 animals treated

○ >10 per 100 animals treated

Which antimicrobial ingredients were used in the farm in the last 6 months?

**x** β-lactam antibiotics (**x** penicillins **x** cephalosporins)

○ tetracyclines

**x** aminoglycosides

○ Polypeptides

○ Macrolides

**x** Fenicols

○ Folic acid antagonists

**x** Chinolons
